# Investigating object detection errors in endoscopic imaging of esophageal SCC and dysplasia through precision–recall analysis

**DOI:** 10.3389/fonc.2025.1707854

**Published:** 2025-12-05

**Authors:** Li-Jen Chang, Kun-Hua Lee, Arvind Mukundan, Riya Karmakar, Achmad Bauravindah, Tsung-Hsien Chen, Chien-Wei Huang, Hsiang-Chen Wang

**Affiliations:** 1 Division of Gastroenterology and Hepatology, Department of Internal Medicine, Ditmanson Medical Foundation Chia-Yi Christian Hospital, Chiayi, Taiwan; 2 Min-Hwei Junior College of Health Care Management, Tainan, Taiwan; 3 Department of Trauma, Changhua Christian Hospital, Changhua, Taiwan; 4 Department of Mechanical Engineering, National Chung Cheng University, Chiayi, Taiwan; 5 School of Engineering and Technology, Sanjivani University, Kopargaon, Maharashtra, India; 6 Department of Computer Science, Universitas Islam Indonesia (UII), Yogyakarta, Indonesia; 7 Department of Internal Medicine, Ditmanson Medical Foundation Chia-Yi Christian Hospital, Chiayi, Taiwan; 8 Department of Nursing, Tajen University, Yanpu, Pingtung, Taiwan; 9 Department of Gastroenterology, Kaohsiung Armed Forces General Hospital, Kaohsiung, Taiwan; 10 Director of Technology Development, Hitspectra Intelligent Technology Co., Ltd., Kaohsiung, Taiwan

**Keywords:** esophageal cancer, hyperspectral imaging, esophageal squamous cell carcinoma, band selection, machine learning, deep learning

## Abstract

**Introduction:**

Esophageal squamous cell carcinoma (ESCC) is difficult to detect early on white-light endoscopy (WLI) because lesions are subtle and artifacts (such as glare, bubbles, text, tools) mimic pathology.

**Methods:**

This study benchmarked five object detectors including two You Only Look Once models (YOLOv5, YOLOv), Faster Region-based Convolutional Neural Networks (Faster R-CNN), Single Shot MultiBox Detector (SSD) and Real-time Detection Transformer (RT-DETR) on WLI dataset using harmonized training (from scratch, 150 epochs, identical hyperparameters) and two label configurations: a 4-label as major categories (SCC, Dysplasia, Bleeding, Inflammation) and an 11-label artifact. Evaluation used macro precision/recall/F1 at IoU 0.50 on a fixed 310-image test set.

**Results:**

Incorporating artifact classes improved overall macro metrics, with YOLOv5/YOLOv8 providing the strongest performance in the 11-label scenarios, however, class-wise findings revealed persistent recall limitations for early disease. In the 11-label analysis, Dysplasia detection remained low (YOLOv5: 88/201, 43.8%; YOLOv8: 82/201, 40.8%), and SCC was only moderate (YOLOv5: 25/44, 56.8%; YOLOv8: 24/44, 54.5%). Confusion analyses showed that errors were dominated by non-detections (“background”) rather than misclassification with benign or artifact labels, while approximately one in five lesion predictions was a spurious unmatched false positive, implicating both sensitivity and specificity constraints.

**Discussion:**

These results indicate that labeling artifacts reduces non-lesion confusion but does not, by itself, recover subtle early lesions. Limitations include single-center, WLI-only data and training from scratch, future work should prioritize endoscopy-specific pretraining, explicit artifact suppression or joint segmentation, and external validation.

## 1 Introduction

Esophageal squamous cell carcinoma (SCC) is one of the most prevalent cancers, as well as its early stage, such as dysplasia. In 2020, globally, there were an estimated 604,100 esophageal cancer cases resulting in 544,100 deaths (1). The American Cancer Society has conveyed the importance of overcoming early cancer detection. The 5-year survival rate is 48% when the cancer is growing only in the esophagus. This number can be as low as 5.4% if metastasized (2). White light imaging (WLI) is one of the methods used to detect esophageal cancer. However, it has low sensitivity and only moderate specificity (40.6% and 76.8%, respectively) in detecting dysplasias and/or chronic esophagitis (3, 4). Several tools that do not solely rely on white light images, e.g., endoscopic ultrasound (EUS), have higher sensitivity (84%) and specificity (99%) (5). Furthermore, narrow band imaging (NBI) has emerged as an effective method, with a sensitivity of 88%, but a specificity of 94%. Despite its high sensitivity, NBI is costly and requires skilled operators (6, 7). Nevertheless, these methods (even the advanced ones such as NBI) are burdened by visually ambiguous features, including inflammation, bubbles, glare, and mucosal folds, that can resemble cancer (8, 9). In addition, virtual artifacts affect approximately 30% of the endoscopic images (10). These challenges make accurate diagnosis more difficult.

Artificial intelligence (AI) is expected to have a significant impact in medical image analysis by 2025 (11–14). Over 340 AI radiology tools have been approved by the Food and Drug Administration (FDA), in particular for clinical use, notably in radiology, where the use of medical images has doubled since 2019 (15). Several studies have adopted deep learning models as their feature extraction and detection tools. You Only Look Once (YOLO) and single-shot multibox detector (SSD) have been proven to be effective in real-time lesion detection (16, 17). In addition, a variant YOLO family model, i.e., CRH-YOLO, as well as YOLOv5 and SSD, has high precision and recall of more than 88% in gastrointestinal endoscopy (18). These models significantly learn and detect complex spatial and texture patterns in images. Nonetheless, subtle features such as early-stage diseases, including dysplasia, are difficult to recognize, yielding high errors of false positives and false negatives. For example, a number of WLI-trained models have less than 50% sensitivity for dysplasia (19, 20). Furthermore, young doctors miss 47% of early SCC cases (21). As an effective clinical tool, these errors erode confidence in the adoption of AI, especially when false alerts can increase the workload in real-world practice.

Early-stage cancer visually resembles benign features such as inflammation (22), glare (23), and mucosal disruption (24). As a result, the features or patterns in the image are difficult to detect. Relying solely on WLI, which has only low-contrast images, can be problematic. Moreover, the image quality issue, which is primarily caused by light interference, can decrease the structural similarity (SSIM) scores by 4% or more (25). On the other hand, the ratio between benign and malignant tumors is 19:1, which can lead to a reduction of 0.066, and thus bias when detected by the model (26). In addition, when the images are used for model training, inconsistent or noisy labels affect the generalizability of the model (27, 28). The majority of research prioritizes accuracy, but ignores the causes of the prediction error. Worryingly, even advanced models use irrelevant demographic information to make the classification report higher, as this is not a standard metric. This highlights the need for the regulation of AI systems, where only 3.6% of approvals report ethnicity/demographic (29).

This study focused on two central research questions. Firstly, what are the specific visual features that mimic the early-stage esophageal cancer lesion analyzed using image feature analysis? Secondly, which label pairs are most often confused, e.g., lesion *vs*. glare and, more specifically, dysplasia *vs*. SCC? Is it due to the dataset being too unbalanced, or having an inconsistent annotation, or having poor image quality? Based on these research questions, this study aimed to investigate the misclassification between features, both SCC and dysplasia, or cancerous features, and benign features such as inflammation, bubbles, and glare. The use of precision and recall can provide a better understanding than just accuracy and is more suitable for imbalanced datasets. Furthermore, confusion matrices were employed to gain more insights. Consequently, this study yields a contribution in the investigation of detection errors in endoscopic imaging of esophageal cancer.

## 2 Methodology

### 2.1 Dataset description

**Figure 1:**
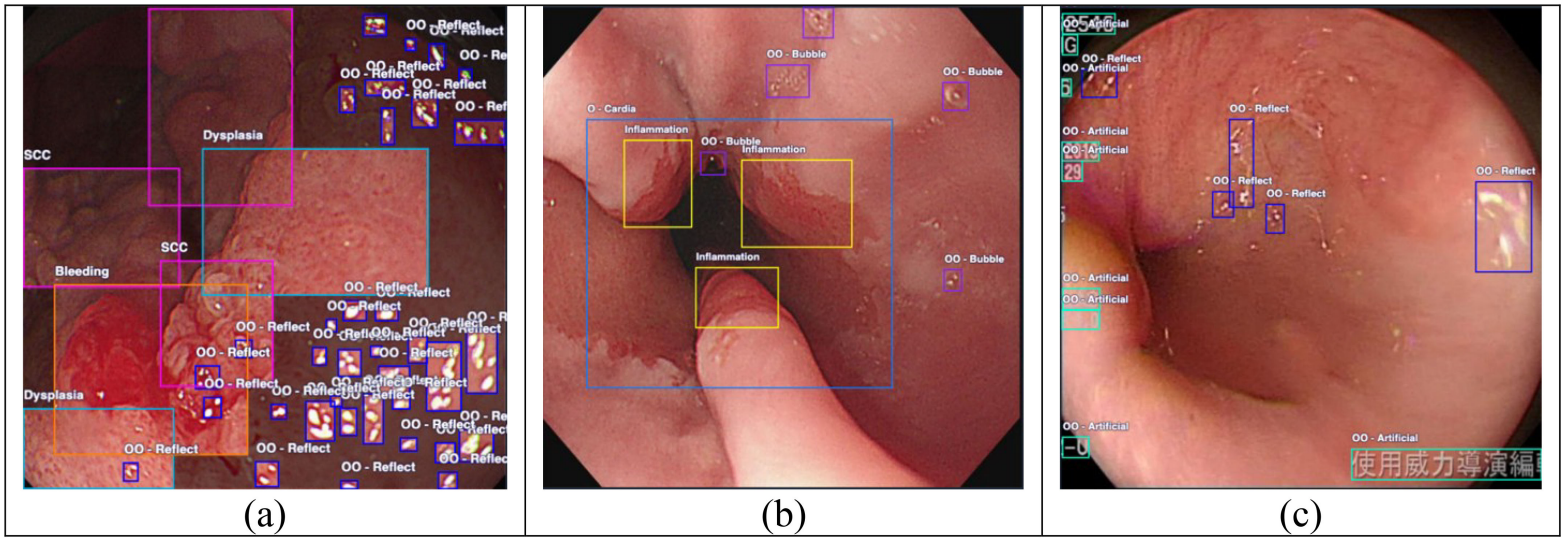
Endoscopy sample images. **(A)** Image consisting of a major category such as dysplasia, squamous cell carcinoma (SCC), and bleeding and showing many specular reflections (*OO-Reflect*). **(B)** Image containing several bubbles (*OO-Bubble*) and non-cancerous objects such as cardia (*O-Cardia*). **(C)** Image with no major category, only artifacts and obstructions such as *OO-Reflect* and text indicators (*OO-Artificial*). *O*, others; *OO*, other objects.

The dataset was acquired from Kaohsiung Medical University Chung-Ho Memorial Hospital, which spans from 2020 to 2021 retrospectively from clinical videos. The endoscopy images were obtained from an Olympus CV-290 extracted from video frames with a resolution of 1,920 × 1,080. Afterward, the obtained files were saved in a .bmp format, which stores the WLI images. To maintain confidentiality, the images, which included sensitive information such as the name, ID, and timestamps, were removed. The images did not have any label or a bounding box (BB), as this study needed, until after labeling performed by a skilled endoscopist using the annotation tool by Tztalin, i.e., LabelImg (30). The sample images are depicted in **Figure 1**. Axis-aligned BBs were employed to delineate all lesions, facilitating standardized training across various object detectors [i.e., YOLOv5/YOLOv8, SSD, faster region-based convolutional neural network (Faster R-CNN), and real-time detection transformer (RT-DETR)]. It has to be recognized that the polygon annotations derived from segmentation can achieve more precision in aligning with the lesion borders and exhibit a reduced labeling impurity, particularly when clinically distinct findings are physically proximate or partially overlapping (e.g., an SCC region and dysplasia). The choice of the BBs aligned with the detector benchmarking focus of this pilot. Nevertheless, polygonal demarcation is more pertinent to clinical practice in specific processes and may be considered in future studies (31).

In this study, each image was annotated into 11 categories based on the endoscopist. Thus, all labels were divided into three main groups: “Major labels,” which represents clinically relevant targets for early detection; “Others,” including non-cancerous, but physiologically normal; and “Other objects,” including those that often visually resemble the pathological regions. To achieve the goals of the study, two separate datasets were used, which both utilized 11 labels within all groups and four labels only in the major category group. This research scenario aids in further analysis and in the investigation of confusion patterns. **Table 1** shows the dataset distribution within the three groups and the 11 labels.

**Table 1:** Dataset distribution.

Group	Label	No. of images
Major label	Dysplasia	1,360
	SCC (squamous cell carcinoma)	230
	Bleeding	384
	Inflammation	132
Others (O)	O-Cardia (esophagogastric junction)	334
	O-Object (exudates, food residues, white spots)	1,417
	O-Through (distal esophageal lumen views)	1,294
Other objects (OO)	OO-Text (artificial information)	751
	OO-Bubble	1,492
	OO-Glare (specular reflections or light glare)	4,654
	OO-Tool (endoscopic tools such as forceps and catheters)	53

O and OO are used to denote “Others” and “Other objects,” respectively, to simplify the representation of categories in the results and discussion.

In order to achieve the goal of the study, several data preprocessing steps were applied. The number of images for each label showed a significant difference, meaning that the dataset was imbalanced. Augmentation was implemented to increase the generalizability and robustness of the model. Both horizontal (50%) (32, 33) and vertical flips (50%) (34, 35) will add synthetic diversity of the images, which will shift the learning approach based on position. Random rotation (from −15° to +15°), which simulates the endoscopy tilt, was applied in order to increase robustness to the viewing angles (36, 37). While applying the augmentation, the image ratio was maintained by providing black padding to the blank rotated pixel. To increase the training time and still keep the features from being overlooked by the model, the image was resized to 640 × 640 (however, 300 × 300 was implemented only to the SSD model).

This dataset composition allows for an in-depth investigation into why AI models may struggle with early cancer detection as the preprocessing technique was initialized to maintain stability across various categories.

### 2.2 Model architecture

To investigate detection errors in early-stage esophageal cancer, we evaluated object detection models, including YOLOv5, YOLOv8, Faster R-CNN, SSD, and RT-DETR. By comparing their handling of visual ambiguities, including glare, bubbles, and low contrast, we aimed to understand how design choices impact performance. The next subsection outlines the structure of each model and the selection rationale.

#### 2.2.1 YOLOv5

YOLOv5, one of the YOLO family of models, is a single-stage detector model. Compared with dual-stage models, its efficiency makes it suitable for real-time scenarios. YOLOv5 consists of a YOLO head for dense prediction across scales of its neck, which is PANet. CSPDarknet is employed for feature extraction. YOLOv5 adopts anchor-based BBs within the sigmoid and binary cross-entropy (BCE) for prediction purposes. It is highlighted that this model uses complete intersection over union (CIoU) for its loss calculation (38). Instead of overlapping between ground-truth and predictions, it applies center distances and aspect ratios with α weighting, as shown in Equation 1 (39). It has been demonstrated that paired white light + hyperspectral (SAVE-enhanced images) improved the diagnosis in multi-institutional compared with RetinaNet on WLI and NBI, with YOLOv5 showing precision and recall of 93.7% and 89.9%, respectively, meaning that it is robust across diverse sources (17). YOLOv5 pairs speed with accuracy. Thus, it is needed in the detection of early esophageal cancer.

(1) $$L_{\text{CIoU}}=1-\text{IoU}+\frac{\rho^{2}(b,b^{gt})}{c^{2}}+\alpha v$$

#### 2.2.2 YOLOv8

YOLOv8 is a later version of the YOLO models, which was established by Ultralytics in early 2023 (40). YOLOv8 uses a similar backbone to YOLOv5, and it replaces the CSPLayer with the C2f module, which is more efficient and performs as a decoupled head separating branches for objectness, classification, and regression. YOLOv8 has a simpler training process and is more adaptable to varying aspect ratios and scales due to its anchor boxes not being predefined, consequently predicting the object centers directly (40). CIoU, focal loss with BCE, and distribution focal loss (DFL) are employed, which retain the model performance in imbalanced detection tasks. DFL, which uses box refinement, replaces a single point of prediction with a probability distribution (40). y the continuous ground-truth offset, while $y_{i}$ two nearest discretized bins, with corresponding predicted probabilities $S_{i}$, are shown $S_{i+1}$, as shown in Equation 2 (41, 42). The loss makes the network assign a high probability to values close to y, allowing the model to express spatial uncertainty. YOLOv8 outperformed in early esophageal cancer, as applied to WLI and spectral-transformed imaging, in terms of precision (19). Its use highlighted its effectiveness in fine-grained differentiation, such as dysplasia and inflammation. Therefore, this can be particularly useful in endoscopic imaging, where the lesion boundaries can be unclear due to blur, occlusion, or inadequate lighting.

(2) $$L_{\text{DFL}}(S_{i},\;S_{i+1})=-[(y_{i+1}-y)\log(S_{i})+(y-y_{i})\log(S_{i+1})]$$

#### 2.2.3 Faster R-CNN

Faster R-CNN is a two-stage detection framework that builds on Fast R-CNN by integrating a region proposal network (RPN) to generate candidate object regions in a unified, end-to-end model, sharing convolutional features for both proposals and detection tasks (43). It typically employs a deep convolutional backbone (such as ResNet), followed by the RPN module to predict the objectness scores and refine the anchor-based BBs and a Fast R-CNN head for the final classification and regression, optimized via a multitask loss combining regression (such as smooth L1) and cross-entropy classification (43). This architecture is praised for producing high-quality region proposals and precise localization, sacrificing inference speed for improved accuracy, making it well suited as a reference benchmark in ambiguous medical image contexts. Faster R-CNN, especially with enhancements, excels in esophageal imaging (44–46). Faster R-CNN uses a multi-task loss that employs a two-part loss function (43), as shown in Equation 3. The first term, $L_{\text{cls}}$, penalizes the misclassification of object proposal $p_{i}^{*}$, while the second, $L_{reg}$, refines the BB coordinates $t_{i}$ using smooth L1 loss. $p_{i}^{*}$ is the ground-truth label (1 for object and 0 for background), and λ balances the classification and localization terms. This approach is powerful in high-precision detection tasks, making it particularly effective in distinguishing visually similar structures such as an inflamed *vs*. a dysplastic tissue.

(3) $$L_{\left(\{p_{i}\},\{t_{i}\}\right)}=\frac{1}{N_{cls}}\sum L_{cls}(p_{i},p_{i}^{*})+\lambda \frac{1}{N_{reg}}\sum p_{i}^{*}L_{reg}(t_{i},t_{i}^{*})$$

#### 2.2.4 Single-shot multibox detector

SSD is a single-stage detection architecture that enables high-speed inference with high accuracy. It predicts object categories and BB adjustments from multiple feature layers in a single pass, eliminating the need for region proposal (47). Its design leverages a backbone network (commonly VGG-16) followed by a bunch of decreasing-scale convolutional feature layers, each predicting default anchor boxes at different scales and aspect ratios. It is optimized jointly using smooth L1 loss for localization and softmax cross-entropy for classification (47). In medical imaging, SSD has demonstrated strong applicability. (48) achieved a mean average precision (mAP) of 95.7% for polyp detection in gastrointestinal endoscopy, outperforming both manual detection and Mask R-CNN and delivering approximately 8.4 times faster inference speeds. Specifically for the detection of esophageal cancer, SSD-based systems achieved a sensitivity up to 0.96 and a specificity of 0.92 in high-definition white light endoscopy (HD-WLE) images, surpassing Faster R-CNN in recall and F-measure in comparative evaluations (49). Furthermore, a hyperspectral imaging (HSI)-enhanced approach using SSD with spectral data highlights its flexibility in integrating multimodal information for early-stage lesion detection in esophageal imaging (50). Therefore, SSD can be a strong candidate for real-time endoscopy-based detection of both dysplasia and esophageal SCC. Equation 4 is the SSD model loss (47), which minimizes a multi-task loss combining classification loss, $L_{\text{conf}}$ (typically softmax cross-entropy), and localization loss, $L_{\text{loc}}$. $x_{i}$ denotes the match indicators, $c_{i}$ the class labels, and $l_{i},g_{i}$ the predicted and ground-truth box coordinates. The coefficient α balances the two terms. This structure allows SSD to predict objects of varying scales from different feature layers, which is ideal for the efficient detection of both small dysplastic lesions and larger SCC regions.

(4) $$L_{\text{SSD}}=\frac{1}{N}\sum_{i}L_{\text{conf}}(x_{i},c_{i})+\alpha \sum_{i}L_{\text{loc}(x_{i},l_{i},g_{i})}$$

#### 2.2.5 RT-DETR

RT-DETR is a single-stage detection architecture that removes the NMS bottleneck by teaching a transformer decoder to output a fixed set of detections in one pass, enabling real-time speed (51). Its number of decoder layers can be trimmed at inference to trade accuracy for latency without retraining. This makes RT-DETR the first DETR family model to hit real-time while matching or outperforming their contemporary YOLO baselines. RT-DETR has been applied in gastrointestinal endoscopy and related workflows. In wireless capsule endoscopy (Kvasir-Capsule), RT-DETR variants reported AP50:95 = 77.8% (RT-DETR-X) with real-time throughput up to ~270 frames per second (FPS) (RT-DETR-S), while the medium model balanced speed and accuracy for practical deployment (52). In esophageal imaging, a study trained five detectors, including RT-DETR, and found that the model has moderate precision (75%) and recall (68%) over conventional WLI on the SCC label (19). Altogether, the end-to-end pipeline of RT-DETR makes it suitable for real-time endoscopic detection of both dysplasia and esophageal SCC. The loss function used in the architecture is shown in Equation 5. Here, $L_{\text{aux}}$ provides dense supervision to the encoder, $L_{o2o}$ enriches one-to-one supervision in the decoder while retaining the end-to-end prediction characteristics, and $L_{o2m}$ supplies one-to-many dense supervision to the decoder. By default, α, β, γ= 1.

(5) $$L=\text{α}L_{\text{aux}}+\text{β}L_{o2o}+\text{γ}L_{o2m}$$

### 2.3 Training configuration

All object detection models were trained using either the Ultralytics framework (for YOLOv5, YOLOv8, and YOLO-NAS) or implemented directly via the PyTorch and TorchVision libraries for other architectures such as SSD and Faster R-CNN. To ensure consistency across model comparisons, this study applied the same training pipeline and hyperparameter settings to all models, unless otherwise specified.

Each model was trained from scratch without using pre-trained weights to prevent bias from pre-learned features unrelated to endoscopic imaging. The training procedure was run for a fixed duration of 150 epochs, without early stopping, to ensure that even the late-stage convergence behavior could be captured. This study used a batch size of 32 and a learning rate of 0.01, optimized using stochastic gradient descent (SGD). The learning rate decay or the scheduling strategies were not applied in the baseline configuration, focusing instead on static learning to evaluate model robustness. The hyperparameters used are shown in **Table 2**. Moreover, the dataset was split into 80% for training, 10% for validation, and 10% for testing. All models were trained using the same data splits to ensure fair and reproducible comparisons. All models were trained for 150 epochs utilizing standardized hyperparameters to ensure comparability of the architectures. It should be recognized that the ideal number of epochs differs among network families: single-stage detectors such as YOLO typically converge more rapidly, while two-stage frameworks such as Faster R-CNN frequently require additional epochs to achieve stability in region proposal optimization.

**Table 2:** Hyperparameters used in the experiment.

Hyperparameter	Value
Framework	Ultralytics, PyTorch, TorchVision
Pre-trained weight	FALSE
Epochs	150
Early stopping	Not used
Batch size	32
Learning rate	0.01
Optimizer	SGD
Dataset split	80%/10%/10%
Learning rate schedule	None

*SGD*, stochastic gradient descent.

### 2.4 Study relevance and workflow

**Figure 2:**
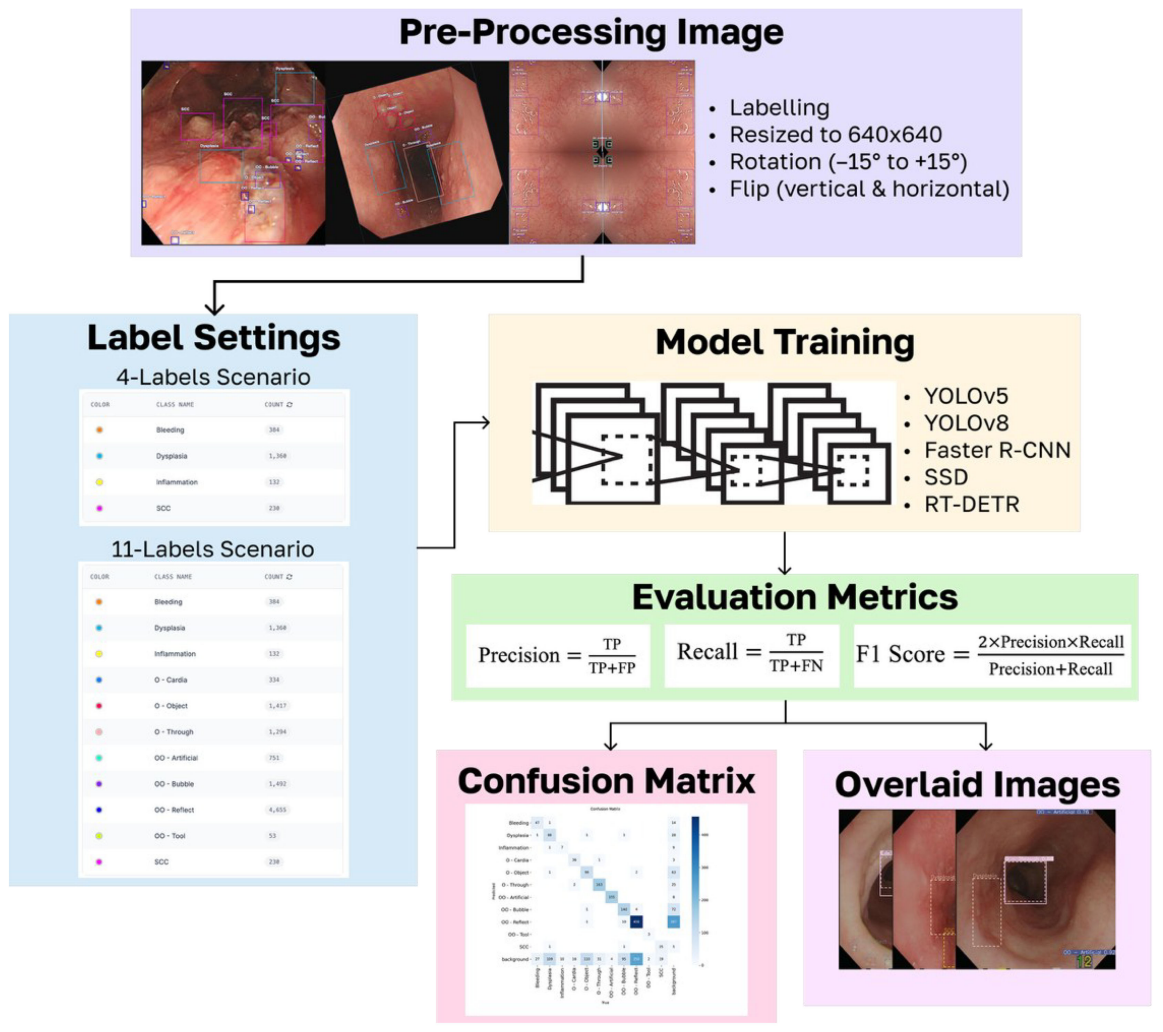
Research workflow. Using several pre-processing images to enhance the generalization of the model, along with two-label settings achieving the study goal, the dataset was trained by five models using several evaluation metrics and a confusion matrix, which then produced overlaid images.

This study evaluated endoscopic object detectors under two label configurations: a four-label setup that focuses on clinically important classes (i.e., SCC, dysplasia, bleeding, and inflammation) and an 11-label setup that introduces realistic visual confounders such as bubbles, glare, cardia, and tools. All models were trained and evaluated using the same data split and preprocessing pipeline, with class-wise performance assessed based on precision, recall, and F1 scores on a fixed test set. This design addresses a key clinical challenge: early esophageal lesions often resemble benign structures, making detection error-prone. The workflow of this study (as shown in **Figure 2**) allows quantifying both the performance and the uncertainty across realistic labeling conditions.

## 3 Results

### 3.1 Overall detection performance

**Figure 3:**
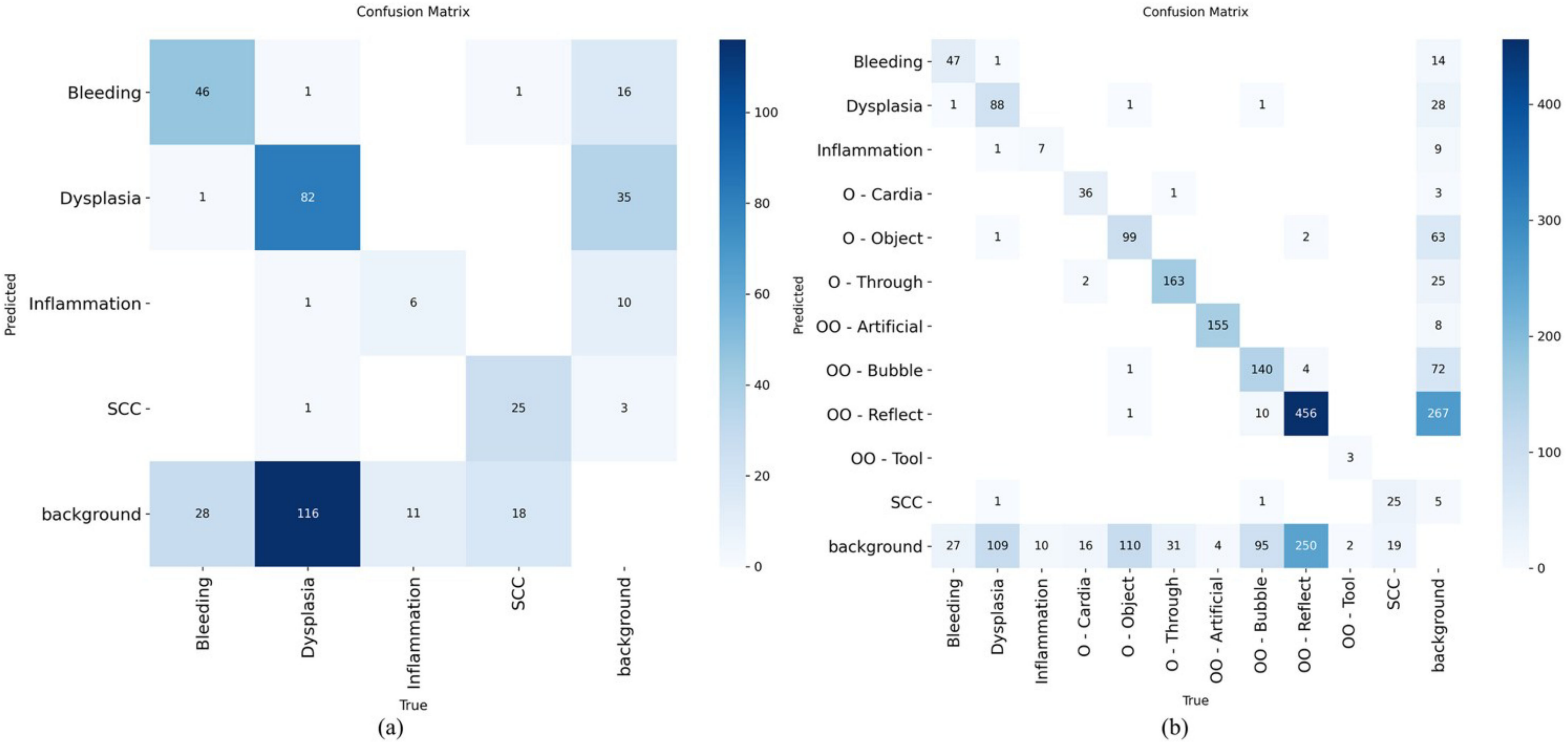
Confusion matrix for YOLOv5 (You Only Look Once version 5) in the four-label setting **(A)**, which highlighted the missed dysplasia dominant, and in the 11-label setting **(B)**, in which the majority of errors occurred as background classification.

**Figure 4:**
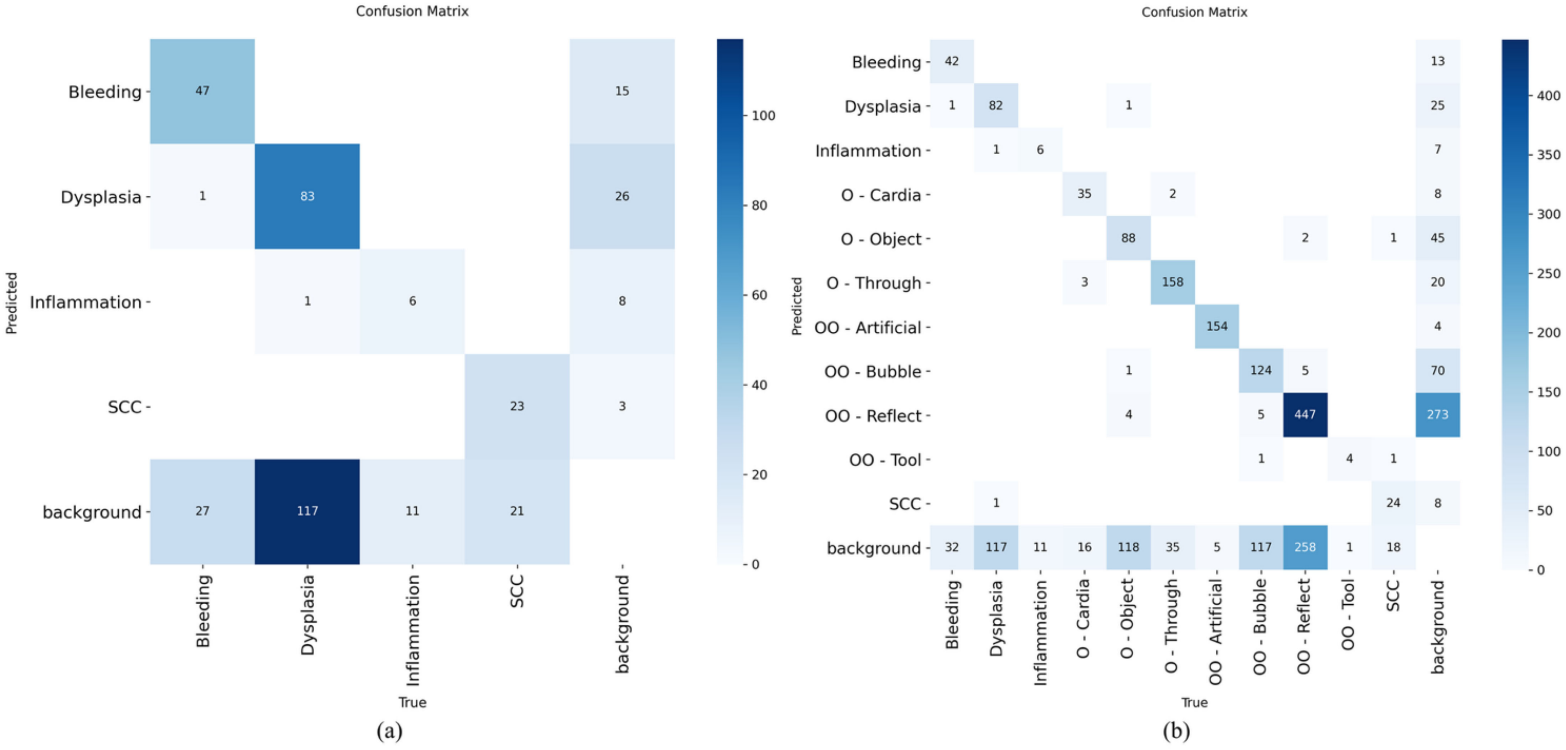
Confusion matrix for YOLOv8 (You Only Look Once version 8) in the four-label setting **(A)**, in which dysplasia was consistently missed as background, and when evaluated on the full 11-label setting **(B)**, which explicitly included artifacts.

Across the five detectors, macro-F1 was higher with the 11-label scenario than with the four-label baseline for every model (median ΔF1 = +0.09). A one-sided paired sign test on the per-model improvements rejected the null of no improvement (*n* = 5, all positive; *p* = 0.031), indicating that the evaluation results are unlikely to be due to chance. Across models and label settings (macro-averaged precision/recall/F1 at IoU = 0.50), as shown in **Supplementary Table S1**, the 11-label configuration consistently outperformed the four-label setup, suggesting that explicitly modeling other malignant (artifact) labels reduces the false positives on clinically relevant targets. Averaged over the five detectors, the macro-precision/recall/F1 improved from 63/43/51 (four-label) to 67/54/60 (11-label). In the four-label scenario, YOLOv5 and YOLOv8 tied on F1 (56 each): YOLOv8 was slightly more precise (76 *vs*. 72), while both reached 45 recall, followed by RT-DETR and Faster R-CNN (F1 = 51 and 48, respectively), with SSD lower (F1 = 43). Under the 11-label scenario, YOLOv5 led overall [F1 = 66, precision/recall (P/R) = 77/58], with YOLOv8 essentially tied (F1 = 65, 73/58). Faster R-CNN showed a lower value (F1 = 60, 66/55), while RT-DETR reached 56 (59/54) and SSD improved to 52 (60/45). All models gained F1 moving from four to 11 labels by +5 to +12 (largest for Faster R-CNN, by +12; YOLOv5 by +10, YOLOv8/SSD by +9, and RT-DETR by +5). These gains were driven primarily by increases in recall (approximately from +9 to 13 across models) with small precision shifts (slight drops for YOLOv8 and RT-DETR, while modest rises for the others). Taken together, the results indicate that detectors benefit from more separated categories that often confuse the model to distinguish. In this discussion, YOLOv5 offered the best overall balance at the macro level in the more realistic both four-label and 11-label settings, with YOLOv8 practically equivalent on F1 (see the confusion matrix in **Figures 3**, **4**).

### 3.2 Class-wise performance

For dysplasia (early cancer), as shown in **Table 3**, for the four-label setting, the best F1 was YOLOv8 at 53 (P/R = 79/39), closely followed by YOLOv5 at 52 (78/39). Faster R-CNN reached 48 (55/42), RT-DETR 47 (64/37), and SSD 42 (47/37). Precision was consistently higher than recall across detectors, underscoring the challenge of recovering dysplasia, which can mimic other objects, with recalls clustered in the 37–42 range. After moving to the 11-label setting, which adds more labels to separate artifacts, the dysplasia F1 scores were 52 (75/40) for YOLOv5, 51 (74/38) for YOLOv8, 50 (64/41) for Faster R-CNN, 45 (56/38) for RT-DETR, and 34 (51/25) for SSD. Relative to the 4-label setting, YOLOv5 remained steady (ΔF1 = 0), Faster R-CNN ticked up slightly (+2) by trading a precision gain (+9) for a small recall decrease (−01), YOLOv8 and RT-DETR slipped slightly (−2 each), and SSD fell notably (−8), driven by a recall jumping down to 25. Averaged across architectures, as shown in **Supplementary Table S2**, the dysplasia F1 decreased from 48 to 46, with recall dropping from 38.8 to 36.5, indicating that the introduction of artifact classes did not, by itself, translate into better early lesion recovery (detailed confusion matrix as shown in **Figures S1**–**S4**).

**Table 3:** Per-class (dysplasia and SCC) metrics across models with different scenarios.

Architecture	Scenario	Label	Precision (%)	Recall (%)	F1 (%)
YOLOv5	Trained with 4 labels	Dysplasia	78	39	52
		SCC	83	52	64
	Trained with 11 labels	Dysplasia	75	40	52
		SCC	88	51	65
YOLOv8	Trained with 4 labels	Dysplasia	79	39	53
		SCC	96	49	65
	Trained with 11 labels	Dysplasia	74	38	51
		SCC	85	52	65
Faster R-CNN	Trained with 4 labels	Dysplasia	55	42	48
		SCC	84	68	75
	Trained with 11 labels	Dysplasia	64	41	50
		SCC	63	57	60
SSD	Trained with 4 labels	Dysplasia	47	37	42
		SCC	68	48	56
	Trained with 11 labels	Dysplasia	51	25	34
		SCC	65	45	53
RT-DETR	Trained with 4 labels	Dysplasia	64	37	47
		SCC	72	45	56
	Trained with 11 labels	Dysplasia	56	38	45
		SCC	64	40	50

*YOLO*, You Only Look Once; *Faster R-CNN*, faster region-based convolutional neural network; SSD, single-shot multibox detector; *RT-DETR*, real-time detection transformer.

For SCC, as shown in **Table 3**, in four labels, Faster R-CNN led SCC, with F1 = 75 (P/R = 84/68), offering the strongest recall. YOLOv8 and YOLOv5 followed at 65 (96/49) and 64 (83/52), respectively, while SSD and RT-DETR were at 56 (68/48) and 56 (72/45), respectively. After expanding to 11 labels, the leadership shifted: YOLOv5 and YOLOv8 tied at F1 = 65, with P/R = 88/51 and 85/52, respectively. Faster R-CNN declined to 60 (63/57), while SSD and RT-DETR showed 53 (65/45) and 50 (64/40), respectively. On average, across models, as shown in **Supplementary Table S3**, the F1 of SCC decreased from 631 to 584, with the precision and recall both lower (P = from 806 to 729, R = from 524 to 492). Thus, while the YOLO variants remained robust on SCC under the richer label space that adds more labels, the two-stage Faster R-CNN lost much from the four-label scenario (from 75 to 60), as well as the transformer-based RT-DETR (from 45 to 40).

The takeaways for the justification of clinic-relevant targets were as follows. 1) Recall showed consistently low metrics results for dysplasia across all detectors, and even with artifact labels, it did not improve and even worsened in some cases, suggesting that subtle and possible to mimic cues still dominate failure modes. 2) For SCC, YOLOv5/YOLOv8 provided the most stable F1 under both label sets, whereas Faster R-CNN excelled only in the simpler label space and was sensitive to label expansion. 3) The precision–recall trade-off is architecture-dependent: as an example, Faster R-CNN gained dysplasia precision with 11 labels, but paid in recall, while SSD struggled the most when the task required distinguishing many classes.

### 3.3 Confusion and misclassification patterns (11-label setting: YOLOv5 *vs*. YOLOv8)

Using extended confusion matrices, as shown in **Figure 3** and **Figure 4**, that included background as both a predicted and a true class, the error for the lesion classes was dominated by non-detections rather than label misclassification with artifacts or other benign findings.

**Figure 5:**
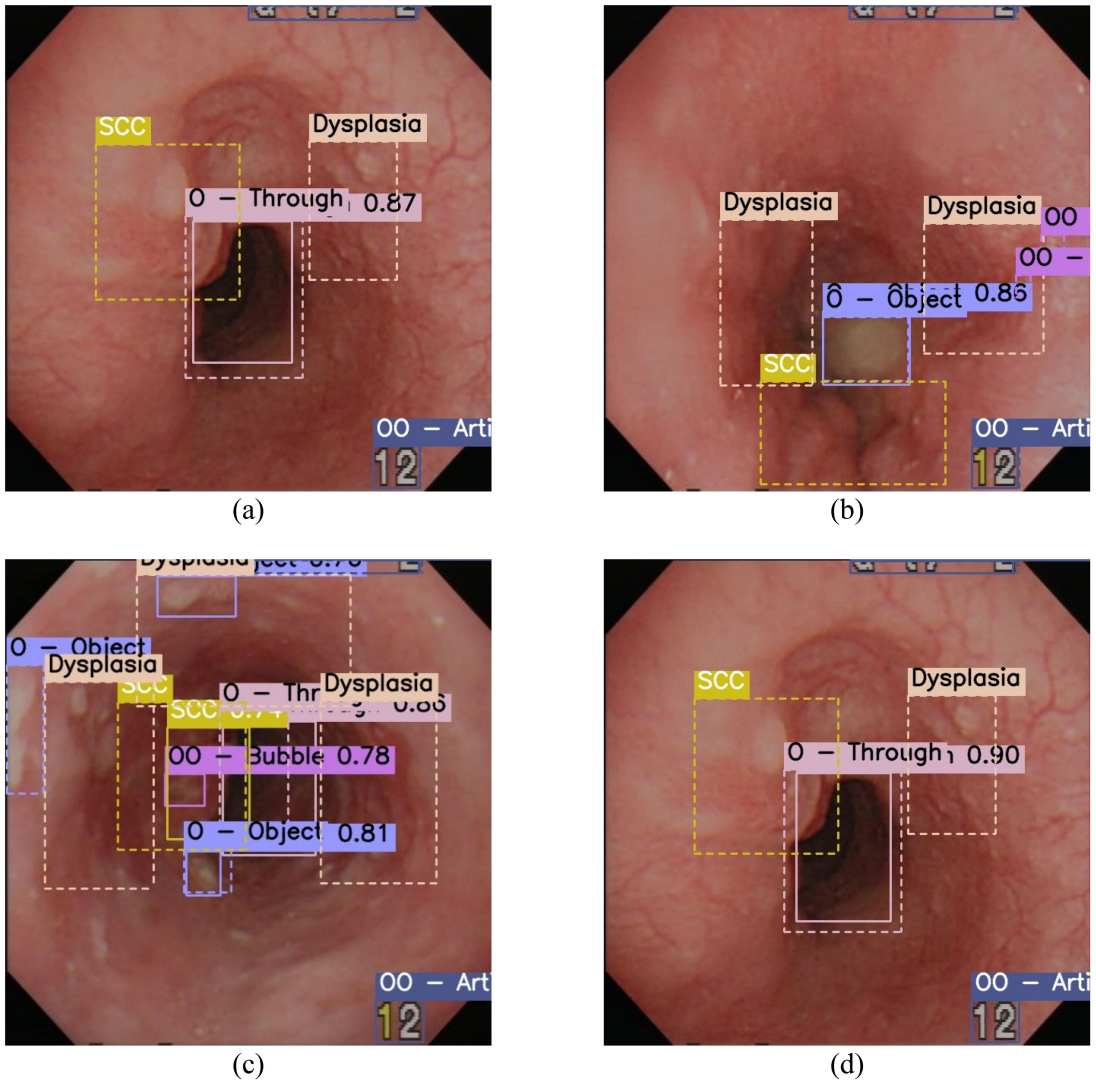
Overlaid images of the predicted (*solid line*) and the actual (*dashed line*) bounding box in the 11-label setting. **(A)** Image produced by YOLOv5 (You Only Look Once version 5), which has five bounding boxes consisting of three labels, where the prediction missed for dysplasia and misclassified for squamous cell carcinoma (SCC). **(B)** YOLOv5 missed all the malignant labels. **(C)** YOLOv8 successfully predicted SCC with 0.74 confidence, while dysplasia was missed. **(D)** Both dysplasia and SCC were missed by YOLOv8.

For dysplasia, with YOLOv5, 88 of 201 dysplasia boxes were correctly detected (43.8%), while 54.2% (109/201) were predicted as background (missed), as depicted in **Figure 5B**. Only 2.0% (4/201) were misclassified into other labels (one each to bleeding, inflammation, O-Object, and SCC, as represented in **Figure 5A**). YOLOv8 showed the same pattern: 82/201 correct (40.8%), 58.2% missed (117/201), both shown in **Figures 5C, D**, and just 1.0% (2/201) misclassified (one to inflammation and one to SCC). In short, >95% of the dysplasia errors were pure misses, not confusion with artifacts. The percentages of the confusion matrices are shown in **Supplementary Table S6**.

For SCC, with YOLOv5, 25 of 44 SCCs were correctly detected (56.8%), with the remaining 43.2% (19/44) missed as background and no cross-label swaps. YOLOv8 detected 24 out of 44 (54.5%) and missed 40.9% (18/44), while only 4.5% (2/44) were misclassified (one to O-Object and one to OO-Tool). Thus, similarly to dysplasia, the SCC errors were predominantly non-detections.

Cross-class misclassifications with artifacts/benign structures were rare for both lesions. For dysplasia, mislabels into non-malignant classes were from one or less to three instances per model; for SCC, these was from none to one instance per artifact class. The key failure was under-detection, not confusion of lesions with glare, bubbles, or other “Others” labels. Therefore, the implication is that these matrices represent where to focus improvements: proposals and sensitivity around subtle lesions (particularly dysplasia), plus the suppression of background false positives for lesion heads.

## 4 Discussion

In a single-center, retrospective WLI esophageal endoscopy, the comparison of five detectors under a four-label *versus* an 11-label scenario, with artifacts added, showed that the addition of explicit artifact classes improved the overall macro performance, but did not uniformly benefit the clinically critical lesion categories. Across models, YOLOv5 and YOLOv8 achieved the strongest macro-level performance in the 11-label setting. However, class-wise analysis highlighted recall for dysplasia as the principal bottleneck: with 11 labels, YOLOv5 had more numbers of missed than misclassifications, similarly with YOLOv8. The confusion matrices demonstrated that the errors were dominated by non-detections (“background”) rather than swaps into artifacts or other benign labels. Approximately one in five lesion predictions were unmatched background false positives, indicating an additional review workload and supporting the need to target high recall. These outcomes align with the dataset characteristics quantified earlier, i.e., substantial artifact presence and a marked class imbalance, both of which make subtle, low-contrast early lesions such as dysplasia difficult to localize. These factors likely suppress small lesions, low-contrast targets, and make the performance sensitive to threshold selection. Methodologically, the findings argue for strategies that increase proposal sensitivity for small and low-contrast lesions in the vicinity of artifacts (such as hard-negative mining around glare, size-aware sampling, and enhanced multiscale features), combined with false-positive control and trustworthy confidence (class-balanced or focal losses, calibration, and the thresholds chosen at a high-recall operating point). For deployment relevance, end-to-end latency at batch 1 on the target hardware (ranging from preprocessing to inference to NMS) needed to be reported, as well as the FPS at 640 × 640 and whether the high-recall operating point sustains near-real-time throughput. Given training from scratch and reporting at IoU of 0.50, absolute values likely sit below the achievable ceiling. Further gains are expected from endoscopy-specific pre-training or self-supervision, explicit artifact suppression or joint segmentation, and evaluation on external cohorts. Overall, the labeling artifacts helps reduce non-lesion confusion, but clinical readiness hinges on closing the dysplasia recall gap and curbing the background false positives rather than on marginal improvements against benign classes. Moreover, to contextualize the workflow impact, further research should be conducted, with analysis on the false positives per frame (unmatched dysplasia/SCC predictions divided by the test frames) and the misses per procedure estimated as lesions per procedure at the chosen operating point, quantifying the alert burden and the missed lesion risk. Several factors contributed to low generalizability and other limitations. Firstly, the evaluation was restricted to WLI. However, clinical utility, which has higher sensitivity and specificity like NBI, was not incorporated. Therefore, performance may differ under NBI or mixed modality, and multimodal training or distillation from NBI to WLI remains untested. Secondly, the cancer type was limited to esophageal SCC, and adenocarcinoma was not included, narrowing the applicability to regions and practices where SCC is prevalent. Thirdly, data were taken only from a single hospital and with a single device generation, which may encode differences in how images were captured. Generalizability can be strengthened by domain adaptation across endoscope models and sites (such as adaptive batch normalization) and by resolution/input scaling ablations (such as 512, 640, and 768) to assess the sensitivity of small lesion recall. Fourthly, no clinical validation was performed, and there were no prospective reader study, no real-time deployment, and no assessment of the workflow impact or alarm fatigue. Therefore, clinical utility cannot be inferred from offline metrics alone. Finally, the work should be interpreted as a pilot study. The sample size and the label scope constraint, as well as the dominant failure mode (missed dysplasia), indicated remaining sensitivity gaps. To address these limitations, future efforts should include multimodal datasets (WLI+NBI and/or HSI), a broader histologic coverage (including adenocarcinoma), multicenter external validation with confidence intervals, and ensemble strategies (such as YOLOv5/YOLOv8 fusion with weighted boxes and test time augmentation) to increase recall while controlling background false positives. Such ensembles should be paired with calibration and thresholding at high-recall operating points and evaluated prospectively for clinical impact. This is a retrospective study based on data from a single institution utilizing solely WLI, which may restrict its generalizability. By systematically incorporating non-dysplasia and artifact labels, it has been demonstrated that contextual annotation of benign and confounding regions can enhance the overall precision of the model and reduce non-lesion misclassification. The aforementioned findings suggest the potential of structured negative labeling as a partial strategy for the development of clinically viable models. Subsequent study will expand this approach to encompass multimodal data, including NBI data and multicenter populations, to ensure uniformity in devices and demographic variations. The primary limitation of the study is the exclusive use of WLI. Clinically, early dysplastic lesions are often assessed using NBI or other improved imaging techniques that provide superior visualization of the mucosal and vascular architecture. The absence of multimodal inputs diminishes the performance of the models on subtle early lesions and restricts their applicability to the overall population. Future work methodology will include multimodal datasets that encompass both WLI and NBI to bridge the modality gap and accurately reflect the diagnostic processes in real-world scenarios. Previous research has shown superior AI performance in early esophageal SCC using enhanced imaging techniques (53). Horie et al. (54) developed a CNN that achieved 98% patient-level sensitivity on a still-image test set, with macro-metric case-level sensitivity, specificity, positive predictive value, and negative predictive value of 77%, 79%, 39%, and 95%, respectively, in mixed WLI environments. In contrast, our benchmark isolates the WLI condition, explicitly models artifacts and benign instances, and demonstrates a macro-metric enhancement in sensitivity. However, it still exhibits a constrained recall rate for dysplasia-specific lesions, as described. Although the YOLO family detectors are increasingly used in gastrointestinal endoscopy, esophageal SCC-specific reports that disclose directly comparable mAP values remain limited; hence, over-interpreting cross-study mAPs was avoided and instead harmonized, same-data comparisons among YOLOv5/YOLOv8 and two-stage baselines were provided within our WLI-only cohort. Despite predictions of enhanced macro measures by artifact identification, our comprehensive study provides pragmatic recommendations for future endeavors: i) class-specific recall profiles, indicating dysplasia as the principal limitation in WLI; ii) confusion matrices and error audits, revealing that non-detections predominantly constitute failures; iii) per-class precision–recall curves and threshold analyses, demonstrating the operation at elevated recall rates; and iv) artifact-inclusive frameworks, aimed at minimizing non-lesion confusion but struggling to identify subtle early lesions. As previously projected, a series of aggregate tendencies were identified. Nevertheless, stratified, class-conscious, and error typological analyses elucidated the points of failure and their underlying causes, offering specific levers to enhance the model and data design. While many results align with the established predictions, the provision of detailed metrics for the classes and types of errors enables comprehensive comparisons across studies, establishing a clear benchmark for the evaluation of multimodal or other externally validated systems in the future. Solely BB-only labels were utilized, which may encompass non-lesional pixels and, in certain cases, include several neighboring discoveries that compromise class purity. This contrasts with polygonal segmentation, which is extensively utilized in specific clinical research contexts and may contribute to the limitations in recalling small lesions, which will be one of the future scopes of the study. Two-stage detectors, such as Mask R-CNN, are theoretically more precise due to the region of interest (RoI) refinement and the segmentation-level supervision. However, the current benchmark did not include these as the dataset was limited to BBs. The two-stage model, i.e., Faster R-CNN, failed to surpass the YOLO models, likely due to the synchronized training regimen and the absence of instance masks, which limited its performance. Future initiatives will enhance the dataset with polygon and instance-mask annotations and will reevaluate segmentation-capable models utilizing Mask R-CNN or YOLO-Seg in multimodal (WLI+NBI) contexts. The consistent 150-epoch duration may not represent the optimal point for each model. One-stage detectors such as YOLO may attain a performance plateau more rapidly and are prone to overfitting during extended training periods, whereas two-stage detectors such as Faster R-CNN can accommodate longer training schedules or phased fine-tuning. Architecture-specific epoch tuning and adaptive early-stopping techniques will be incorporated subsequently to obtain a more equitable convergence rate and generalization in future studies.

## 5 Conclusion

Early detection of esophageal SCC remains a critical clinical need, which imposes a substantial global burden (~604,100 cases and 544,100 deaths in 2020), and the outcomes are strongly dependent on the stage. Moreover, the 5-year survival is ~48% when confined to the esophagus, but only ~5% after metastasis. Furthermore, the standard WLE is prone to missed subtle lesions, and advanced imaging such as NBI is not always available. This study benchmarked five object detectors on WLI esophageal endoscopy using harmonized training and two label configurations (four clinical labels *vs*. an 11-label, artifact-added configuration). The incorporation of artifact classes improved the overall macro-precision/recall/F1. However, the clinically critical targets, i.e., dysplasia and SCC, remained recall-limited, and confusion analyses showed errors dominated by non-detections rather than the swaps with benign/artifact classes. These findings indicate that artifact labeling reduces non-lesion confusion, but does not, by itself, recover subtle early lesions. Clinical readiness therefore hinges on increasing the proposal sensitivity for small, low-contrast lesions near glare and other confounders while curbing background false positives, such as size-aware sampling, hard-negative mining around artifacts, stronger multi-scale features, class-balanced/focal losses, calibration, and thresholds set at high-recall operating points. Generalizability is constrained by key limitations: training from scratch, metrics at IoU of 0.50, WLI-only imaging without NBI (modality gap), the inclusion of only SCC among the esophageal cancer types (histology gap), single-hospital demography and device context (site/hardware bias), the absence of prospective clinical validation (workflow impact unknown), and the pilot-scale scope. Near-term gains are likely from ensemble strategies (such as YOLOv5/YOLOv8 fusion) to boost recall while controlling false positives, whereas longer-term progress should incorporate multimodal datasets including NBI, broader histologic coverage, multicenter external validation with confidence intervals, and prospective reader or real-time studies. Within the current constraints, YOLOv5/YOLOv8 under the 11-label schema offer the strongest baselines, but deployment should await improvements that close the dysplasia recall gap and reduce spurious alerts.

## Data Availability

The original contributions presented in the study are included in the article/**Supplementary Material**. Further inquiries can be directed to the corresponding authors.
