# Correction: Investigating object detection errors in endoscopic imaging of esophageal SCC and dysplasia through precision–recall analysis

**DOI:** 10.3389/fonc.2025.1773175

**Published:** 2026-01-06

**Authors:** Li-Jen Chang, Kun-Hua Lee, Arvind Mukundan, Riya Karmakar, Achmad Bauravindah, Tsung-Hsien Chen, Chien-Wei Huang, Hsiang-Chen Wang

**Affiliations:** 1 Division of Gastroenterology and Hepatology, Department of Internal Medicine, Ditmanson Medical Foundation Chia-Yi Christian Hospital, Chiayi, Taiwan; 2 Min-Hwei Junior College of Health Care Management, Tainan, Taiwan; 3 Department of Trauma, Changhua Christian Hospital, Changhua, Taiwan; 4 Department of Mechanical Engineering, National Chung Cheng University, Chiayi, Taiwan; 5 School of Engineering and Technology, Sanjivani University, Kopargaon, Maharashtra, India; 6 Department of Computer Science, Universitas Islam Indonesia (UII), Yogyakarta, Indonesia; 7 Department of Internal Medicine, Ditmanson Medical Foundation Chia-Yi Christian Hospital, Chiayi, Taiwan; 8 Department of Nursing, Tajen University, Yanpu, Pingtung, Taiwan; 9 Department of Gastroenterology, Kaohsiung Armed Forces General Hospital, Kaohsiung, Taiwan; 10 Director of Technology Development, Hitspectra Intelligent Technology Co., Ltd., Kaohsiung, Taiwan

**Keywords:** esophageal cancer, hyperspectral imaging, esophageal squamous cell carcinoma, band selection, machine learning, deep learning

Affiliation 9 was erroneously given as

“Department of Medical Research, Dalin Tzu Chi Hospital, Buddhist Tzu Chi Medical Foundation, Chiayi, Taiwan”. The correct affiliation is “Department of Gastroenterology, Kaohsiung Armed Forces General Hospital, Kaohsiung, Taiwan”.

The original version of this article has been updated.

