# Supplementary material for: Investigating object detection errors in endoscopic imaging of esophageal SCC and dysplasia through precision–recall analysis
Source: Front Oncol. 2025 Dec 5;15:1707854. doi: 10.3389/fonc.2025.1707854 (PMC12714927; doi:10.3389/fonc.2025.1707854)
Supplement: Supplementary file 1 [file DataSheet1.pdf]

frontiers

Supplementary Material

1 Evaluation Matrix

1.1 Precision

Precision measures how many of the predicted positive cases for a given class are truly correct. A high precision for dysplasia implies the model rarely mistakes benign features (e.g., glare or folds) for early cancer. This is essential to reduce alarm fatigue among clinicians. It is defined in Equation 1.Supplementary material is not typeset so please ensure that all information is clearly presented, the appropriate caption is included in the file and not in the manuscript, and that the style conforms to the rest of the article.

$$\text{Precision} = \frac{\text{TP}}{\text{TP+FP}} \quad (1)$$

Where:

TP = True Positives (correct detections of the class)

FP = False Positives (incorrect detections labeled as the class)

1.2 Recall

Recall (or Sensitivity) measures how many actual positive cases are successfully detected by the model. High recall for ESCC or dysplasia means the model detects the majority of true cancer cases, critical for minimizing missed diagnoses, especially in early-stage conditions. It is defined in Equation 2.

$$\text{Recall} = \frac{\text{TP}}{\text{TP+FN}} \quad (2)$$

1.3 F1 Score

The harmonic mean of precision and recall, providing a single score to evaluate model performance, especially for imbalanced classes like dysplasia. It is defined in Equation 3.

$$\text{F1 Score} = \frac{2\times\text{Precision}\times\text{Recall}}{\text{Precision+Recall}} \quad (3)$$

2 Results

Table 1. Overall Performance Across Models Using Macro-Average

Overall Performance Across Models				
**Architecture**	**Scenario**	**Precision**	**Recall**	**F1**
**YOLOv5**	4 Labels	0.72	0.45	0.56
	11 Labels	0.77	0.58	0.66
**YOLOv8**	4 Labels	0.76	0.45	0.56
	11 Labels	0.73	0.58	0.65
**Faster-RCNN**	4 Labels	0.51	0.45	0.48
	11 Labels	0.66	0.55	0.60
**SSD**	4 Labels	0.54	0.36	0.43
	11 Labels	0.60	0.45	0.52
**RT - DETR**	4 Labels	0.62	0.43	0.51
	11 Labels	0.59	0.54	0.56
**All Models (Average)**	4 Labels	0.63	0.43	0.51
	11 Labels	0.67	0.54	0.60

Table 2. Comparison of 4 and 11 labels averaged dysplasia of across models

Scenario	Label	Architecture	Precision	Recall	F1
Trained with 4 Labels	Dysplasia	**YOLOv5**	0.777	0.388	0.518
		**YOLOv8**	0.794	0.393	0.526
		**Faster-RCNN**	0.549	0.420	0.476
		**SSD**	0.472	0.373	0.417
		**RT - DETR**	0.638	0.368	0.467
Average			0.646	0.388	0.481
Trained with 11 Labels	Dysplasia	**YOLOv5**	0.750	0.398	0.520
		**YOLOv8**	0.741	0.383	0.505
		**Faster-RCNN**	0.643	0.409	0.500
		**SSD**	0.505	0.254	0.338
		**RT - DETR**	0.560	0.383	0.455
Average			0.640	0.365	0.464

Table 3. Comparison of 4 labels and 11 labels averaged SCC of across models

Scenario	Label	Architecture	Precision	Recall	F1
Trained with 4 Labels	SCC	**YOLOv5**	0.833	0.523	0.642
		**YOLOv8**	0.956	0.490	0.647
		**Faster-RCNN**	0.844	0.675	0.750
		**SSD**	0.677	0.477	0.560
		**RT - DETR**	0.638	0.368	0.467
Average			0.790	0.507	0.613
Trained with 11 Labels	SCC	**YOLOv5**	0.750	0.398	0.520
		**YOLOv8**	0.741	0.383	0.505
		**Faster-RCNN**	0.643	0.409	0.500
		**SSD**	0.677	0.477	0.560
		**RT - DETR**	0.718	0.455	0.557
Average			0.706	0.424	0.528

Table 4. Row-normalized lesion confusion (counts and rates)

Model	GT	Pred: Dysplasia (count)	Pred: SCC (count)	Pred: Others (count)	Pred: Missed (count)	GT total (count)	Pred: Dysplasia (rate)	Pred: SCC (rate)	Pred: Others (rate)	Pred: Missed (rate)
YOLOv5	Dysplasia	88	1	3	109	201	0.4378	0.005	0.0149	0.5423
	SCC	0	25	0	19	44	0	0.5682	0	0.4318
YOLOv8	Dysplasia	82	1	1	117	201	0.408	0.005	0.005	0.5821
	SCC	0	24	2	18	44	0	0.5455	0.0455	0.4091

Table 5. Prediction-side composition for Dysplasia/SCC (counts and rates)

Model	Pred class	Total predictions (count)	TP (count)	Misclass→Others (count)	Background FP (count)	TP (rate)	Misclass→Others (rate)	Background FP (rate)
YOLOv5	Dysplasia	115	85	2	28	0.7391	0.0174	0.2435
YOLOv5	SCC	30	0	23	6	0	0.7667	0.2
YOLOv8	Dysplasia	109	82	2	25	0.7523	0.0183	0.2294
YOLOv8	SCC	33	24	0	8	0.7273	0	0.2424

Figure 1. Confusion Matrix of Faster RCNN

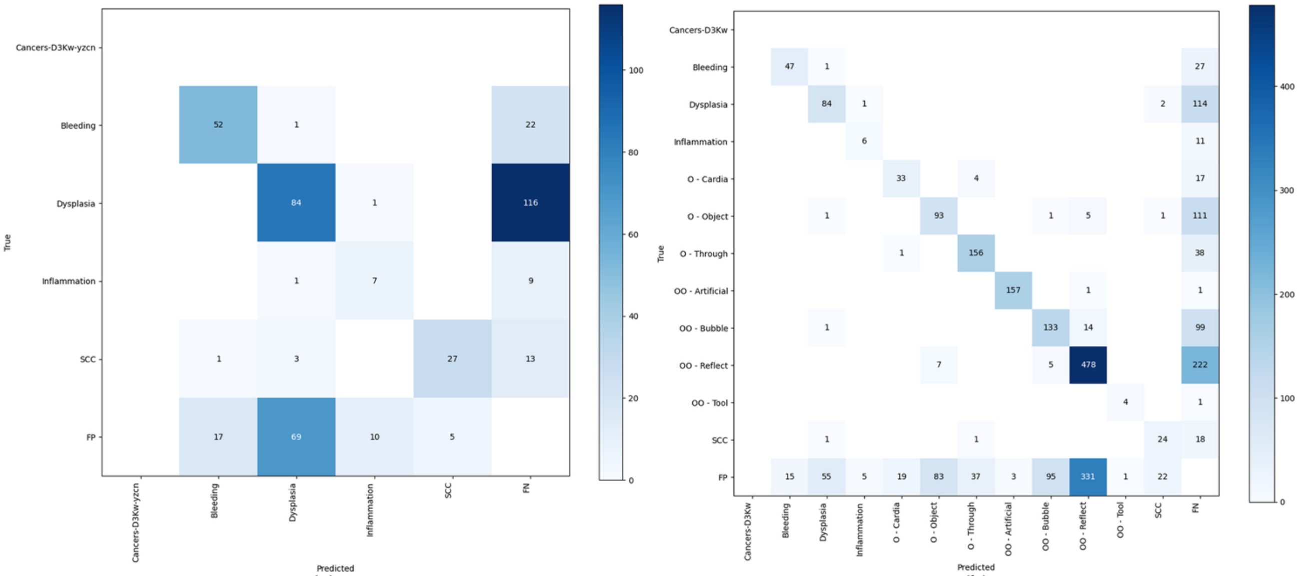

(a) (b)

Figure 2. Confusion Matrix of SSD

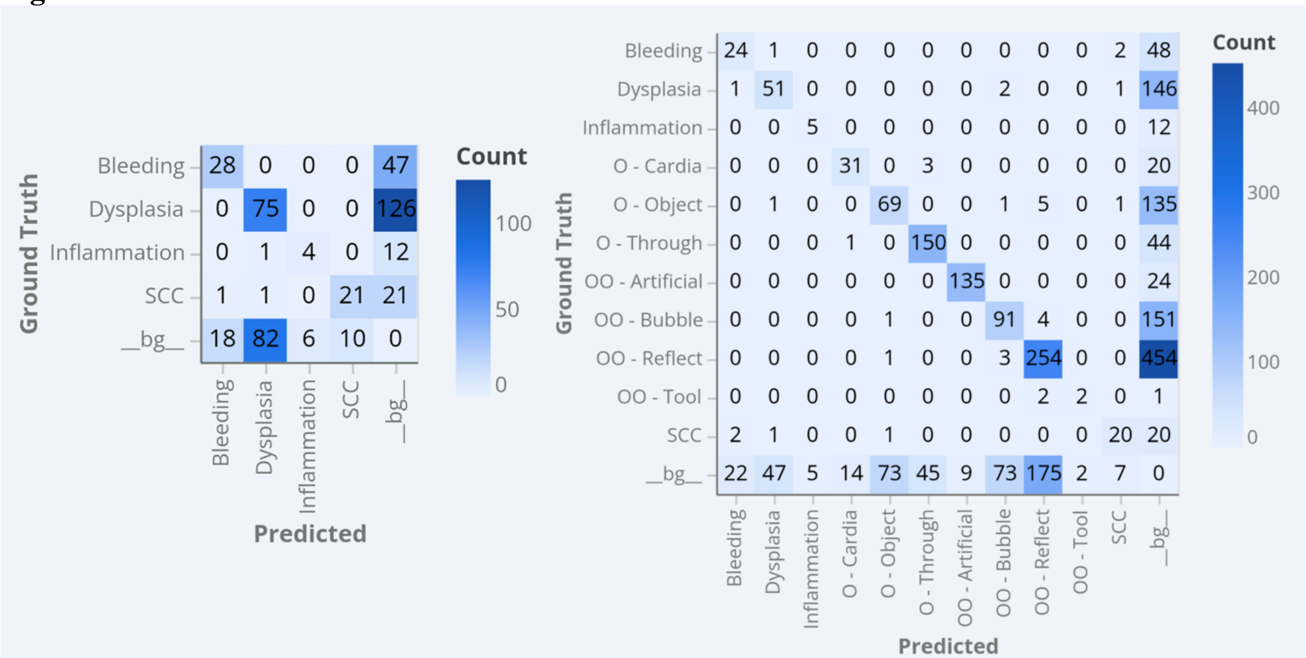

(a) (b)

Figure 3. Confusion Matrix of RT DETR

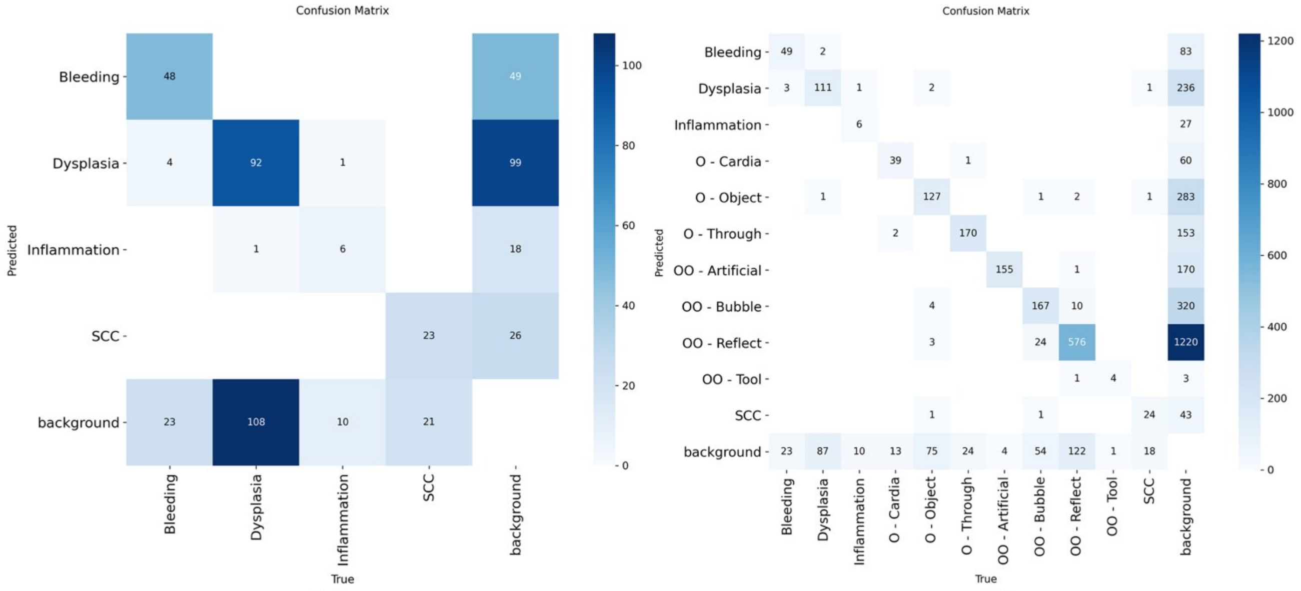

(a) (b)

Figure 4. YOLO Architecture

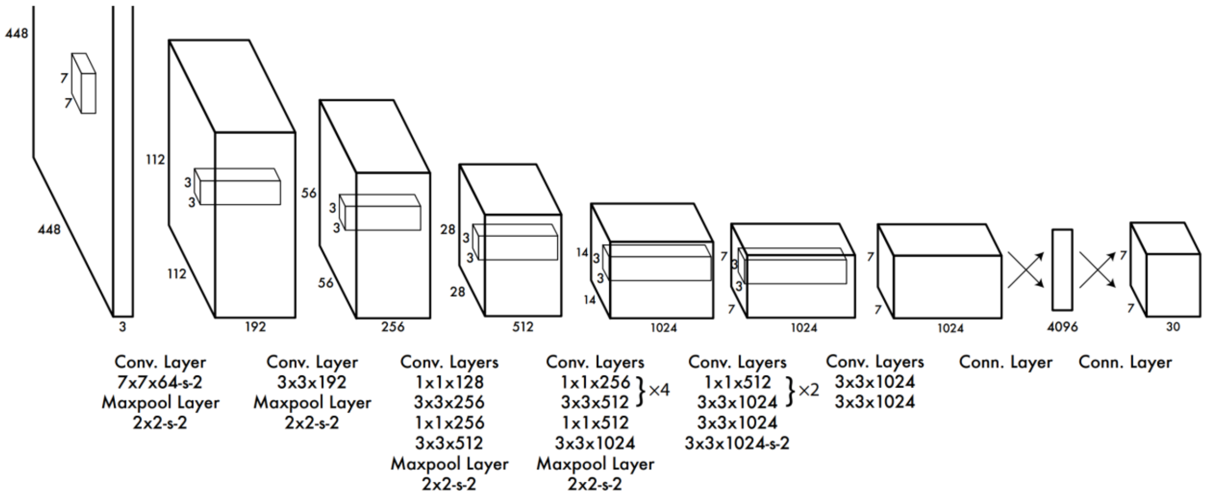

Figure 5. Faster R-CNN Architecture

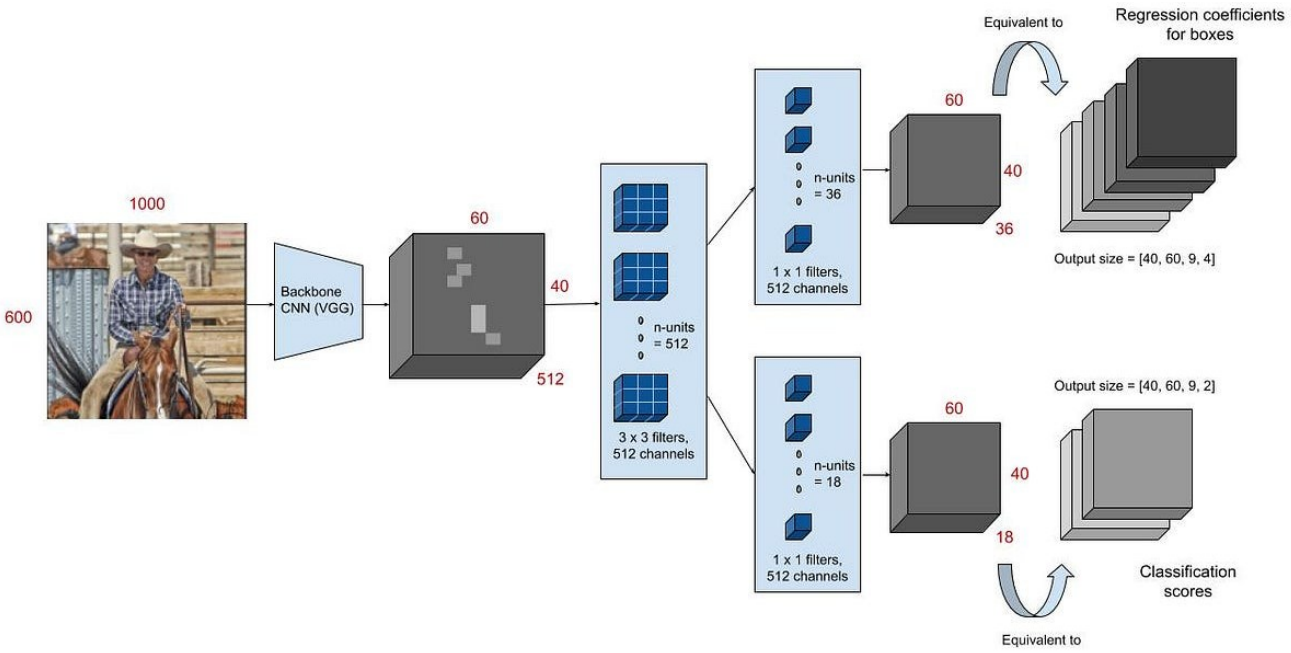
Equivalent to
Regression coefficients for boxes
60
40
36
n-units = 36
1 x 1 filters, 512 channels
Output size = [40, 60, 9, 4]
1000
600
Backbone CNN (VGG)
60
40
512
n-units = 512
3 x 3 filters, 512 channels
Output size = [40, 60, 9, 2]
60
40
18
n-units = 18
1 x 1 filters, 512 channels
Classification scores
Equivalent to
